# Time–Frequency Characteristics of Vehicle–Bridge Interaction System for Structural Damage Detection Using Multi-Synchrosqueezing Transform

**DOI:** 10.3390/s25144398

**Published:** 2025-07-14

**Authors:** Mingzhe Gao, Xinqun Zhu, Jianchun Li

**Affiliations:** School of Civil and Environmental Engineering, University of Technology Sydney, Ultimo, NSW 2007, Australia; mingzhe.gao@student.uts.edu.au (M.G.); jianchun.li@uts.edu.au (J.L.)

**Keywords:** time-frequency characteristics, vehicle-bridge interaction, muti-synchrosqueezing transform, bridge health monitoring, time-frequency representation, concrete bridges

## Abstract

Structural damage in bridges is typically a local phenomenon. When a vehicle passes over the damage location, it induces a local response, which is highly sensitive to the damage. The interaction between the bridge and moving vehicle is a non-stationary time-varying process. The local damage can be accurately identified by analyzing the time-varying characteristics of the bridge response subjected to a moving vehicle. Synchrosqueezing transform, a reassignment method used to sharpen time–frequency representations, offers an effective tool to decompose the non-stationary signal into distinct components. This paper proposes a novel method based on multi-synchrosqueenzing transform to extract the time-varying characteristics of the vehicle–bridge interaction systems for bridge structural health monitoring. A vehicle–bridge interaction model is built to simulate the bridge under moving vehicles. Different damage scenarios of concrete bridges have been simulated. The effect of bridge damage parameters, the vehicle speed, the road surface roughness on the time-varying characteristics of the vehicle–bridge interaction system is studied. Numerical and experimental results demonstrate that the proposed method efficiently and accurately extracts the time-varying features of the vehicle–bridge interaction system, which could serve as potential indicators of structural damage in bridges.

## 1. Introduction

Bridges are deteriorated due to aging and operational and environmental loads, especially under traffic loadings. Structural health monitoring (SHM) is an effective method to detect structural damage at the early stage for bridge safety [1]. Bridge damage is a local phenomenon. The vehicle passing over the damage location induces a local response and it is very sensitive to the local damage [2]. Recent vehicle–bridge interaction (VBI)-based SHM has attracted the interest of researchers and practical engineers [3]. This dynamic interaction offers valuable insights into a bridge’s structural health but also introduces challenges due to the nonlinear nature of structural damage and the transient nature of vehicle movement.

A moving vehicle passing over the bridge is a time-varying process. It is crucial to understand the dynamic properties of VBI systems for predicting the behavior of bridge structures under moving vehicles. Jalili and Esmailzadeh [4] presented the dynamic VBI effect with the bridge simplified as a simply supported Euler–Bernoulli beam. Zhong et al. [5] analyzed the impact of prestress on the dynamic responses of prestressed bridges under moving vehicles and the bridge was modeled as a continuous beam with eccentric prestress. Zhu and Law [6] presented the inverse problems of VBI dynamics and moving force identification, and structural parameter identification with the moving load are two typical inverse problems. Chen et al. [7] presented the dynamic coupling effect in steel–concrete bridges under heavy vehicular loads, and tire characteristics were discussed in detail, such as nonlinear suspension and tire–ground contact characteristics. When there is damage to the bridge, it exhibits the complex nonlinear behavior of the bridge under moving vehicles. Fanning et al. [8] conducted service load testing on masonry arch bridges and found that nonlinear properties under service load could be an indicator of structural capacity. Law and Zhu [9] presented the dynamic behavior of damaged reinforced concrete bridge structures under moving vehicular loads. Zhang et al. [10] revealed local nonlinearities from beam-to-beam bolted connections and initial damage during the destructive field testing of a reinforced concrete bridge. Yin et al. [11] investigated the effect of the bridge damage on the vibration of the vehicle–bridge coupled system, such as cracks, and a massless rotational spring was used to describe the local flexibility due to the crack damage. These studies highlight the complex nonlinear properties of bridge systems due to damage. The dynamic response of the damaged bridge subjected to moving vehicles is nonlinear and non-stationary. It is essential to extract time-varying features from the dynamic responses of the bridge under moving vehicles for bridge condition assessment.

Advanced signal processing techniques are useful to extract bridge coupled system time-varying features of non-stationary signals. Short-time Fourier transform (STFT) and continuous wavelet transform (CWT) are two of the most typical techniques for time–frequency analysis, and time-varying characteristics of the signal are obtained by transforming a one-dimensional time series into a two-dimensional time–frequency representation (TFR) [12]. These linear TFR methods are limited by the Heisenberg uncertainty principle to obtain high time and frequency resolutions in parallel [13]. The conventional time–frequency analysis methods, such as STFT, CWT, and Hilbert–Huang transform, cannot provide comprehensive insights into the dynamic characteristics of vehicle–bridge interaction systems due to their limited resolution [14]. To overcome these limitations, synchrosqueezing transform (SST) and its variants, such as the STFT-based SST [15] and CWT-based SST [16,17], have been introduced to sharpen the TFR of the signal. These advanced SST algorithms can enhance the resolution and accuracy of time–frequency analysis, providing better diagnostics for structural health. Liu et al. [16] presented a time-varying analysis method for structural damage detection using CWT-based SST. Sony and Saghu [18] combined SST with multivariate empirical mode decomposition to identify the time-varying structural system. The dynamic response of the bridge under a passing vehicle is non-stationary. The change in bridge responses due to a vehicle passing over the damage location can be captured for bridge damage detection by time–frequency analysis. Li et al. [19] presented a synchroextracting transform (SET)-based method to extract the time-varying characteristics of bridges under the passage of vehicles. Tang et al. [14] introduced the frequency-reassignment operator from the local maximum SST into the absolute value of the generalized S transform to extract the time-varying characteristics of the bridge responses under operational traffic loads for structural damage detection. The above transform algorithms cannot adequately address close modes and strongly time-varying signals [20]. To address these challenges, Multi-Synchrosqueezing Transform (MSST) was proposed to improve the energy concentration of the SST method through an iterative procedure [21]. MSST was used to identify the modal parameters of civil structures [22]. Li et al. [23] investigated the time-varying frequencies of VBI systems using MSST and offered a more granular analysis of time-varying characteristics in VBI systems. As shown above, MSST consists of multiple operations of SST and it offers a more concentrated time–frequency representation compared with SET and SST, which allows a more accurate decomposition of the non-stationary signal.

This paper proposes an innovative method based on MSST to extract the time-varying characteristics of VBI systems for bridge damage detection. The VBI system is simulated using the finite element model, and the dynamic responses of the bridge subjected to a moving vehicle are obtained. The time-varying characteristics are extracted from the dynamic responses of the bridge using the proposed method. The effects of the vehicle–bridge mass and frequency ratios, road surface roughness, and the vehicle speed on time-varying characteristics are studied. Different damage scenarios of concrete bridges are simulated. The change pattern of the time-varying characteristics due to the damage is discussed. Numerical simulations and experimental results show that the proposed method is effective and accurate in extracting time-varying characteristics for structural damage detection. This paper is organized as the theory of MSST and SST in Section 2 and a numerical study in Section 3. In Section 4, an experimental study is conducted to verify the proposed method, and the conclusions are summarized in Section 5.

## 2. Theory

The preliminary theory regarding the time-varying dynamic interaction between the vehicle and bridge is first introduced. A new Multi-Synchrosqueezing Transform (MSST)-based method is proposed to extract the time-varying characteristics of the vehicle–bridge interaction (VBI) system in this section.

### 2.1. Vehicle–Bridge Interaction Systems

#### 2.1.1. VBI Modeling

**Figure 1:**
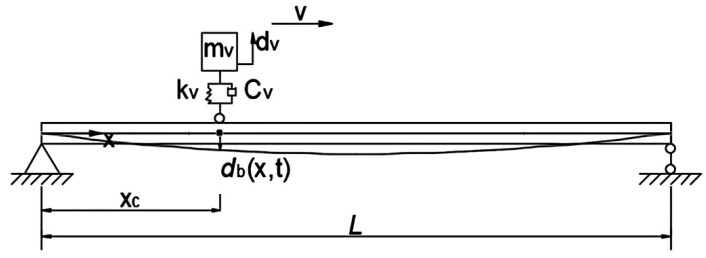
Vehicle–bridge interaction systems.

As shown in Figure 1, a vehicle–bridge system is represented as a continuous uniform bridge subjected to a moving vehicle. For the vehicle–bridge interaction analysis, common vehicle models include the quarter-vehicle, half-vehicle and full three-dimensional vehicle models. This study’s aim is to develop a method to extract the time-varying characteristics from the dynamic responses of a beam bridge under a moving vehicle. The vertical motion of the vehicle’s center of mass and the tire point is the key factor. So the single-axle vehicle model is adopted in this study. The vehicle is assumed to travel along the longitudinal direction of the bridge at a constant speed v. The simply supported bridge is modeled using the finite element model. The Equations of motion for the bridge and vehicle subsystems can be expressed as follows [19]:

(1) $$m_{v}{\ddot{d}}_{v}(t)+c_{v}{\dot{d}}_{v}(t)+k_{v}d_{v}(t)=P_{vint}(t)$$

(2) $$M_{b}{\ddot{d}}_{b}(t)+C_{b}{\dot{d}}_{b}(t)+K_{b}d_{b}(t)=H_{c}(t)P_{\mathrm{bint}\;}(t)$$

where ${\ddot{d}}_{v},\;{\dot{d}}_{v},\;d_{v}$ are the vertical acceleration, velocity, and displacement responses of the vehicle, respectively. ${\ddot{d}}_{b},\;{\dot{d}}_{b},\;d_{b}$ denote the vertical acceleration, velocity, and displacement responses of the bridge, respectively. $M_{b}$, $C_{b}$, $K_{b}$ are the mass, damping, and stiffness matrices of the bridge, respectively. $P_{vint}\left(t\right)=k_{v}d_{cp}(t)+c_{v}{\dot{d}}_{cp}(t)$ is the force to the vehicle subsystem at the contact point between the vehicle and bridge. $d_{cp}\left(t\right)=d_{b}\left(x\left(t\right),\;t\right)+r(x\left(t\right))$ is the contact displacement between the vehicle and bridge at the location x(t). $r\left(x\right)$ is the road surface roughness. $P_{\mathrm{bint}\;}\left(t\right)=m_{v}g-P_{vint}.$ $P_{b\mathrm{int}\;}(t)$ is the interacting force for the vehicle to the bridge. $H_{c}\left(t\right)={\left\{0,\;0,\;\ldots ,\;H_{i}\left(t\right),\;\ldots ,0\right\}}^{T}$ is a Hermitian cubic interpolation function of time. $H_{i}(t)$ is the vector of the shape function in the *ith* element on which the moving vehicle is located at time instant *t* as

(3) $$H_{i}\left(t\right)={\left\{1-3{\xi \left(t\right)}^{2}+2\xi \left(t\right),\left(\xi \left(t\right)-2{\xi \left(t\right)}^{2}+{\xi \left(t\right)}^{3}\right)l_{e},3{\xi \left(t\right)}^{2}-2{\xi \left(t\right)}^{3},{\left(-{\xi \left(t\right)}^{2}+{\xi \left(t\right)}^{3}\right)l}_{e}\right\}}^{T}$$

where $\xi (t)=\left(x(t)-(i-1)l_{e}\right)/l_{e}$, (i−1)$l_{e}\leq x(t)\leq il_{e}$. $l_{e}$ is the length of the element.

#### 2.1.2. Non-Stationary Dynamic Characteristics of the VBI System

Combining Equations (1) and (2), the equation of motion for the VBI system can be obtained as

(4) $$\begin{array}{ll} \; & \left[\begin{array}{cc} M_{b} & m_{v}H_{c}\\ 0 & m_{v} \end{array}\right]\left\{\begin{array}{c} {\ddot{d}}_{b}\\ {\ddot{d}}_{v} \end{array}\right\}+\left[\begin{array}{cc} C_{b} & 0\\ -c_{v}H_{c}^{T} & c_{v} \end{array}\right]\left\{\begin{array}{c} {\dot{d}}_{b}\\ {\dot{d}}_{v} \end{array}\right\}+\left[\begin{array}{cc} K_{b} & 0\\ -k_{v}H_{c}^{T}-c_{v}{\dot{H}}_{c}^{T} & k_{v} \end{array}\right]\left\{\begin{array}{c} d_{b}\\ d_{v} \end{array}\right\}\\ \; & =\left\{\begin{array}{c} H_{c}m_{v}g\\ k_{v}r(x)+c_{v}vr^{\prime }(x) \end{array}\right\} \end{array}$$

where r(x) is the surface roughness profile of the bridge. In Equation (4), the system matrices of the VBI system are time-dependent, determined by the location of the vehicle on the bridge, and the system’s frequencies vary over time. The dynamic responses of the VBI system can be obtained by solving Equation (4).

To understand the time-varying characteristics of the VBI system, the bridge is considered as a uniform Euler beam. Assuming ${\ddot{d}}_{v}\ll g$, the VBI system becomes uncoupled. The analytical solutions of instantaneous frequencies for the vehicle and bridge are given as shown below [24]. When the natural frequency of the vehicle $\omega_{v0}$ is larger than the first natural frequency of the bridge $\omega_{b0}$, the analytical solution is

(5) $$\begin{array}{ll} \omega_{v}^{2}\left(t\right) & =\frac{1}{2}\left(\omega_{v0}^{2}+\omega_{b0}^{2}\right)+\frac{m_{v}\omega_{v0}^{2}}{m_{b}}\sin^{2}\left(\frac{\pi x_{c}\left(t\right)}{L}\right)\\ & +\sqrt{\sqrt{{\left(\frac{\omega_{v0}^{2}}{2}+\frac{\omega_{b0}^{2}}{2}+\frac{m_{v}\omega_{v0}^{2}}{m_{b}}{sin}^{2}\left(\frac{\pi x_{c}(t)}{L}\right)\right)}^{2}-4\omega_{v0}^{2}\omega_{b0}^{2}}}\\ \omega_{b}^{2}\left(t\right) & =\frac{1}{2}\left(\omega_{v0}^{2}+\omega_{b0}^{2}\right)+\frac{m_{v}\omega_{v0}^{2}}{m_{b}}\sin^{2}\left(\frac{\pi x_{c}\left(t\right)}{L}\right)\\ & -\sqrt{\sqrt{{\left(\frac{\omega_{v0}^{2}}{2}+\frac{\omega_{b0}^{2}}{2}+\frac{m_{v}\omega_{v0}^{2}}{m_{b}}{sin}^{2}\left(\frac{\pi x_{c}(t)}{L}\right)\right)}^{2}-4\omega_{v0}^{2}\omega_{b0}^{2}}} \end{array}$$

When the natural frequency of the vehicle $\omega_{v0}$ is smaller than the first natural frequency of the bridge $\omega_{b0}$, the analytical solution is

(6) $$\begin{array}{ll} \omega_{v}^{2}\left(t\right) & =\frac{1}{2}\left(\omega_{v0}^{2}+\omega_{b0}^{2}\right)+\frac{m_{v}\omega_{v0}^{2}}{m_{b}}\sin^{2}\left(\frac{\pi x_{c}\left(t\right)}{L}\right)\\ & -\sqrt{\sqrt{{\left(\frac{\omega_{v0}^{2}}{2}+\frac{\omega_{b0}^{2}}{2}+\frac{m_{v}\omega_{v0}^{2}}{m_{b}}\sin^{2}\left(\frac{\pi x_{c}\left(t\right)}{L}\right)\right)}^{2}-4\omega_{v0}^{2}\omega_{b0}^{2}}}\\ \omega_{b}^{2}\left(t\right) & =\frac{1}{2}\left(\omega_{v0}^{2}+\omega_{b0}^{2}\right)+\frac{m_{v}\omega_{v0}^{2}}{m_{b}}\sin^{2}\left(\frac{\pi x_{c}\left(t\right)}{L}\right)\\ & +\sqrt{\sqrt{{\left(\frac{\omega_{v0}^{2}}{2}+\frac{\omega_{b0}^{2}}{2}+\frac{m_{v}\omega_{v0}^{2}}{m_{b}}{sin}^{2}\left(\frac{\pi x_{c}(t)}{L}\right)\right)}^{2}-4\omega_{v0}^{2}\omega_{b0}^{2}}} \end{array}$$

where $\omega_{v}$ is the instantaneous frequency of the vehicle. $\omega_{b}$ is the instantaneous frequency of the bridge. $m_{v}$ is the mass of the vehicle. $m_{b}$ is the mass of the bridge. L is the length of the bridge. $x_{c}(t)$ is the position of the vehicle on the bridge as a function of time.

#### 2.1.3. Damage Simulation of Reinforced Concrete Bridges

Concrete bridges are widely constructed. For a reinforced concrete beam bridge, the damage is modeled by a decrease in the flexural rigidity of the beam as [25]

(7) $$EI\left(x\right)=E_{0}I\left(1-\alpha {cos}^{2}\left(\frac{\pi }{2}{\left(\frac{\left|x-l_{c}\right|}{\beta L/2}\right)}^{m}\right)\right),\;\left(l_{c}-\beta L/2<x<l_{c}+\beta L/2\right)$$

Here, *E*_0_ and *I* represent the modulus of elasticity and the second moment of area for the beam when it is undamaged. The parameters α, β, and *m* are used to quantify the magnitude, length, and flexural rigidity variation in the damage zone. The parameter *l_c_* indicates the central point of the damage zone, measured from the beam’s left support. The dimensionless parameter β is defined as the ratio between the length of the damage zone and the whole length of the beam, with values ranging from 0.0 to 1.0. The β value of 1.0 means the damage over the whole beam length. Parameter α measures the severity of the damage, varying from 0.0 to 1.0. An α value of 0.0 indicates that the beam remains undamaged, whereas an α value of 1.0 implies a complete loss of flexural rigidity at the midpoint of the damage zone. The parameter *m* reflects the change in the flexural rigidity across the damage zone, from the center to its ends. The damage becomes a flat pattern when m is larger than 1. The details are referred to in the reference [25]. In this study, different damage scenarios are simulated in concrete beam bridges using Equation (7).

### 2.2. Time-Varying Feature Extraction Using Synchrosqueezing Transform

#### 2.2.1. Synchrosqueezing Transform (SST)

Short-time Fourier transform (STFT)-based synchrosqueezing transform is used in this study. For a given response $s(t)\in L^{2}(R)$, it is the response of the bridge subjected to a moving vehicle, as shown in Equations (1) and (2). The STFT of the response with a window function $g(t)\in L^{2}(R)$ is defined as

(8) $$G(t,\omega )=\int_{-\infty }^{+\infty }g(\tau )s(t+\tau )e^{-i\omega \tau }d\tau$$

where the window g(t) compactly supports in $\left[-\Delta_{t},\Delta_{t}\right]$.

For a non-stationary multicomponent signal s(t), it can be expressed as the superposition of the intrinsic mode functions (IMFs) as

(9) $$s\left(t\right)=\sum_{k=1}^{N}A_{k}(t)e^{i\varphi_{k}(t)}$$

where $A_{k}\left(t\right),\;\varphi_{k}(t)$ are the amplitude and phase of the *kth* IMF, respectively. For a slow varying signal, there exists a small enough value ε, $\left|{A\prime }_{k}\left(t\right)\right|\leq \varepsilon ,\left|{\varphi ”}_{k}(t)\right|\leq \varepsilon \forall t$. Here, ${A^{\prime }}_{k}(t)$ and ${\varphi^{”}}_{k}(t)$ are the first derivatives of the signal’s amplitude function $A_{k}\left(t\right)$ and the second derivatives of the phase function $\varphi_{k}(t)$, respectively. Under this assumption, each IMF can be approximated as a purely harmonic signal in a short time. According to the Taylor expansion, the *kth* IMF can be expressed as

(10) $$x_{k}\left(t+\tau \right)=A_{k}\left(t+\tau \right)e^{i\varphi_{k}\left(t+\tau \right)}\approx A_{k}\left(t\right)e^{i\left(\varphi_{k}\left(t\right)+{\varphi^{\prime }}_{k}\left(t\right)\tau \right)}\;for\;\tau \approx 0$$

Substituting Equation (10) into Equation (8), it can be written as follows:

(11) $$\begin{array}{ll} G(t,\omega ) & =\sum_{k=1}^{N}\int_{-\infty }^{+\infty }g(\tau )A_{k}(t)e^{i\left(\varphi_{k}(t)+{\varphi_{k}}^{\prime }(t)\tau \right)}e^{-i\omega \tau }d\tau \\ \; & =\sum_{k=1}^{N}A_{k}(t)e^{i\varphi_{k}(t)}\int_{-\infty }^{+\infty }g(\tau )e^{-i\left({{\omega -\varphi }_{k}}^{\prime }\left(t\right)\right)\tau }d\tau \\ \; & =\sum_{k=1}^{N}A_{k}\left(t\right)e^{i\varphi_{k}\left(t\right)}\hat{g}\left(\omega -{\varphi_{k}}^{\prime }\left(t\right)\right)=\sum_{k=1}^{N}G_{k}(t,\omega ) \end{array}$$

where $\hat{g}\left(\omega \right)$ is the Fourier transform of g(t), $\hat{g}\left(\omega \right)\;\in [-\Delta \omega ,\Delta \omega ]$. Then the derivative with respect to time for G(t,ω) can be obtained as

(12) $$\begin{array}{ll} \partial_{t}G(t,\omega ) & =\sum_{k=1}^{N}\partial_{t}\left(A_{k}(t)e^{i\varphi_{k}(t)}\hat{g}\left(\omega -{\varphi \prime }_{k}(t)\right)\right)\\ \; & \approx \sum_{k=1}^{N}A_{k}(t)e^{i\varphi_{k}\left(t\right)}\hat{g}\left(\omega -{\varphi^{\prime }}_{k}\left(t\right)\right)i{\varphi \prime }_{k}(t)\\ \; & =\sum_{k=1}^{N}G_{k}(t,\omega )i{\varphi^{\prime }}_{k}(t) \end{array}$$

For which $G_{k}(t,\omega )$≠0, the instantaneous frequency (IF) ${\hat{\omega }}_{k}(t,\omega )$ can be obtained by

(13) $${\hat{\omega }}_{k}\left(t,\omega \right)={\varphi^{\prime }}_{k}\left(t\right)=\frac{\partial_{t}G_{k}(t,\omega )}{iG_{k}(t,\omega )}$$

Assuming ε is sufficiently small, let $\tilde{\varepsilon }$= $\varepsilon^{1/3}$, and for $|G_{k}\left(t,\omega \right)|\geq \tilde{\varepsilon }$, it can be approximate as [5,6]

(14) $$\left|{\hat{\omega }}_{k}(t,\omega )-{\varphi^{\prime }}_{k}(t)\right|\leq \tilde{\varepsilon }$$

Equation (10) indicates that for a weakly time-varying signal, ${\hat{\omega }}_{k}(t,\omega )$ can be approximated as the IF ${\varphi^{\prime }}_{k}(t)$ of the signal. Synchrosqueezing transform (SST) uses a frequency-reassignment operator to collect the time–frequency (TF) coefficient.

(15) $$sst(t,\omega_{k})=\int_{-\infty }^{+\infty }G(t,\omega )\delta (\omega_{k}-\hat{\omega }(t,\omega ))d\omega$$

Using SST, the diffuse energy present in the STFT result can be concentrated into a compact region around the IF trajectories of each mode. This essentially enhances the time–frequency representation of the signal.

#### 2.2.2. Multi-Synchrosqueezing Transform (MSST)

SST can provide a time–frequency analysis for signals when the signal is well-separated with slow variations. Compared with STFT, SST can yield a clear time–frequency representation. When the signal variation is large, SST may not be able to provide an accurate and clear time–frequency representation [26]. Multi-synchrosqueezing transform (MSST) is used to execute multiple SST operations for achieving an even sharper time–frequency representation as [21]

(16) $$\begin{array}{ll} \; & {sst}^{\left[2\right]}(t,\omega_{0})=\int_{-\infty }^{+\infty }s{st}^{\left[1\right]}(t,\omega )\delta (\omega_{0}-\hat{\omega }(t,\omega ))d\omega \\ \; & {sst}^{\left[3\right]}(t,\omega_{0})=\int_{-\infty }^{+\infty }s{st}^{\left[2\right]}(t,\omega )\delta (\omega_{0}-\hat{\omega }(t,\omega ))d\omega \\ \; & \;\vdots \\ \; & {sst}^{\left[N\right]}(t,\omega_{0})=\int_{-\infty }^{+\infty }s{st}^{\left[N-1\right]}(t,\omega )\delta (\omega_{0}-\hat{\omega }(t,\omega ))d\omega \end{array}$$

where N is the iteration number, and N > 1. With Equation (16), SST can be executed multiple times to achieve the accurate time–frequency representation iteratively. As an example, N = 2, and MSST can be expressed as

(17) $$\begin{array}{ll} {Ts}^{\left[2\right]}\left(t,\omega_{0}\right) & =\int_{-\infty }^{+\infty }Ts^{\left[1\right]}\left(t,\xi \right)\delta \left(\omega_{0}-\hat{\omega }\left(t,\xi \right)\right)d\xi \\ & =\int_{-\infty }^{+\infty }\int_{-\infty }^{+\infty }G\left(t,\omega \right)\delta \left(\xi -\hat{\omega }\left(t,\omega \right)\right)\times d\omega \delta \left(\omega_{0}-\hat{\omega }\left(t,\xi \right)\right)d\xi \\ & =\int_{-\infty }^{+\infty }G\left(t,\omega \right)\int_{-\infty }^{+\infty }\delta \left(\xi -\hat{\omega }\left(t,\omega \right)\right)\times \delta \left(\omega_{0}-\hat{\omega }\left(t,\xi \right)\right)d\xi d\omega \\ & =\int_{-\infty }^{+\infty }G\left(t,\omega \right)\delta \left(\omega_{0}-\hat{\omega }\left(t,\hat{\omega }\left(t,\omega \right)\right)\right)d\omega \end{array}$$

The same as SST in Section 2.2.1, the IF estimation $\hat{\omega }(t,\hat{\omega }(t,\omega ))$ can be obtained. For a general assumption, there exists ε, sufficiently small, such that |A’(t)|≤ε and |φ’’’(t)|≤ε for all *t*. The signal can be approximately considered as the linear chirp signal in a short time. The expressions are written as $A\left(t+\tau \right)=A(t)$ and $\varphi \left(t+\tau \right)=$ $\varphi (t)+\varphi^{\prime }(t)(\tau -t)+0.5\varphi^{\prime \prime }(t)(\tau -t{)}^{2}$, where terms of order O(A’(t)) and O(φ’’’(t)) are neglected. Therefore, the signal s(t) can be rewritten as

(18) $$s(t+\tau )=A(t)e^{i\left(\varphi (t)+\varphi^{\prime }(t)(\tau -t)+0.5\varphi^{\prime \prime }(t)(\tau -t{)}^{2}\right)}$$

According to Equation (14), a window function is set as the Gaussian function $g(t)=e^{-0.5t^{2}}$. The STFT of the signal is obtained as

(19) $$\begin{array}{c} G\left(t,\omega \right)=\int_{-\infty }^{+\infty }e^{-0.5(\tau -t{)}^{2}}A\left(t\right)e^{i\left(\varphi (t)+\varphi^{\prime }(t)(\tau -t)+0.5\varphi^{\prime \prime }(t)(\tau -t{)}^{2}\right)}\times e^{-i\omega \left(\tau -t\right)}d\tau \\ =A\left(t\right)e^{i\varphi \left(t\right)}\int_{-\infty }^{+\infty }e^{-0.5\left(1-i\varphi^{\prime \prime }\left(t\right)\right)(\tau -t{)}^{2}}e^{-i\left(\omega -\varphi^{\prime }\left(t\right)\right)\left(\tau -t\right)}\times d\left(\tau -t\right)\\ =A\left(t\right)e^{i\varphi \left(t\right)}\frac{1}{\sqrt{1-i\varphi^{\prime \prime }\left(t\right)}}e^{-\frac{\left(\omega -\varphi^{\prime }(t{)}^{2}\right)}{2\left(1-i\varphi^{\prime \prime }(t)\right)}} \end{array}$$

Substituting Equation (19) into Equation (13), the IF of the signal can be obtained as

(20) $$\begin{array}{rr} \hat{\omega }(t,\omega )= & \varphi^{\prime }(t)+\frac{\varphi^{\prime \prime }(t{)}^{2}}{1+\varphi^{\prime \prime }(t{)}^{2}}\left(\omega -\varphi^{\prime }(t)\right)-i\frac{\varphi^{\prime \prime }(t)}{1+\varphi^{\prime \prime }(t{)}^{2}}\left(\omega -\varphi^{\prime }(t)\right)\\ \; & \; \end{array}$$

The real part of IF is as [27]

(21) $$\hat{\omega }(t,\omega )=\varphi^{\prime }(t)+\frac{\varphi^{\prime \prime }(t{)}^{2}}{1+\varphi^{\prime \prime }(t{)}^{2}}\left(\omega -\varphi^{\prime }(t)\right)$$

It can be observed that $\hat{\omega }\left(t,\omega \right)$ cannot provide an unbiased estimation of the true IF. The difference between $\hat{\omega }\left(t,\omega \right)$ and $\varphi^{\prime }(t)$, i.e., |$\hat{\omega }\left(t,\omega \right)-\varphi^{\prime }\left(t\right)|$ is dependent on two factors: the second-order derivative of the instantaneous phase (IP) and the distance between the frequency variable and the true IF. For the strong frequency-modulated signals, the term φ″(t) cannot be neglected, which leads to |$\hat{\omega }\left(t,\omega \right)-\varphi^{\prime }\left(t\right)|$ becoming increasingly larger with the growing distance between the frequency variable and the true IF. This is the reason why the original SST cannot generate a concentrated time–frequency representation for strongly time-varying signals.

According to Equation (17), as an example N = 2, and the IF can be estimated by MSST as

(22) $$\begin{array}{ll} \hat{\omega }(t,\hat{\omega }(t,\omega )) & =\varphi^{\prime }(t)+\frac{\varphi^{\prime \prime }(t{)}^{2}}{1+\varphi^{\prime \prime }(t{)}^{2}}\left(\hat{\omega }(t,\omega )-\varphi^{\prime }(t)\right)\\ \; & =\varphi^{\prime }(t)+{\left(\frac{\varphi^{\prime \prime }(t{)}^{2}}{1+\varphi^{\prime \prime }(t{)}^{2}}\right)}^{2}\left(\omega -\varphi^{\prime }(t)\right) \end{array}$$

In general, MSST can be expressed as

(23) $$Ts^{\left[N\right]}(t,\omega_{0})=\int_{-\infty }^{+\infty }G(t,\omega )\delta \left(\omega_{0}-{\hat{\omega }}^{\left[N\right]}(t,\omega )\right)d\omega$$

Setting a window function as the Gaussian function, the corresponding IF estimate by MSST can be obtained as

(24) $${\hat{\omega }}^{\left[N\right]}(t,\omega )=\varphi^{\prime }(t)+{\left(\frac{\varphi^{\prime \prime }(t{)}^{2}}{1+\varphi^{\prime \prime }(t{)}^{2}}\right)}^{N}\left(\omega -\varphi^{\prime }(t)\right)$$

#### 2.2.3. IF Extraction Using Ridge Detection

Ridge detection is normally used to find the best frequency curve Ω(t) from the time–frequency representation (TFR). Brevdo et al. [27] presented a technique to identify the optimal frequency curve, Ω(t) from TFR $S_{x}$, and it maximizes the energy under a smoothness constraint enforced through a total variation penalization term as [28]

(25) $$\overset{ˆ}{\Omega }=\underset{\Omega }{argmax}\int_{R}{\left|S_{x}(t,\Omega (t))\right|}^{2}\;dt-\lambda \int_{R}{\left|\frac{d\Omega }{dt}(t)\right|}^{2}\;dt$$

### 2.3. Validation of MSST

To show the performance of MSST for time–frequency analysis, the following non-stationary signal is used as an example.

(26) $$x\left(t\right)=\cos\left(2\pi *\left(0.1t^{2.6}+3\sin\left(2t\right)+10t\right)\right)+e^{-0.2t}cos(2\pi *\left(40+t^{1.3}\right)t)$$

As shown in Equation (26), there are two components in the signal with the amplitudes and phases as $A_{1}\left(t\right)=1$, $\varphi_{1}\left(t\right)=0.1t^{2.6}+3\sin\left(2t\right)+10t$ and $A_{2}\left(t\right)=e^{-0.2t}$, and $\varphi_{2}\left(t\right)=\left(40+t^{1.3}\right)t$, respectively. The IFs of these two components are ${\varphi^{\prime }}_{1}\left(t\right)=2.6t^{1.6}+6\cos\left(2t\right)+10$ and ${\varphi^{\prime }}_{2}\left(t\right)=40+2.3t^{1.3}$, respectively.

**Figure 2:**
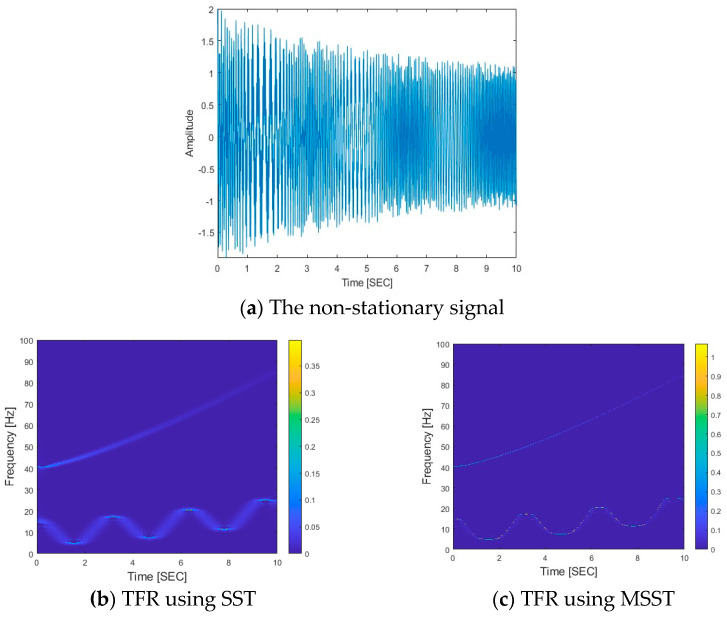
A non-stationary signal and its TFRs using SST and MSST.

The non-stationary signal and its TFR are shown in Figure 2. Figure 2a shows the non-stationary signal, and its TFRs using SST and MSST are shown in Figure 2b,c, respectively. From Figure 2b,c, there are two clear components. One is a time-varying harmonic frequency component and another one is an exponential change frequency component. These two components correspond to two IFs. The results show that the IFs of the signal are successfully extracted using SST and MSST. Compared with the SST results in Figure 2b, that by MSST in Figure 2c provides a quite sharp and much clearer representation of these two components. The results show that MSST has a superior performance in TFR. A high degree of signal concentration typically correlates with reduced time–frequency cross-terms, yielding clearer and more precisely defined time–frequency characteristics. The enhanced concentration by MSST is attributed to complex or optimized algorithmic mechanisms employed during processing, such as improved time–frequency resolution or refined techniques for the separation of frequency components. MSST is shown to more effectively suppress mode mixing and background noise, enhancing the signal’s localization in the time–frequency domain, which is particularly crucial for the analysis of complex non-stationary signals. In this study, MSST is used to extract the time-varying characteristics of VBI systems for structural damage detection.

## 3. Numerical Study for Extracting Time-Varying Characteristics of VBI Systems

### 3.1. Time-Varying Characteristics of the VBI System

A simply supported beam bridge is used as an example in this study. The length, mass density, and flexural rigidity of the bridge are L = 30 m, ρ = 6000 kg/m, and EI = 2.5 × 10^10^ Nm^2^, respectively. The bridge is discretized into 10 beam elements. The Rayleigh damping is considered in this study as C_b_ = α_1_M_b_ + α_2_K_b_, where α_1_ is 0.243 and α_2_ is 0.0001. M_b_, C_b_, and K_b_ denote the mass, damping, and stiffness matrices of the bridge, respectively. In practice, the damping coefficients could be estimated using the first two frequencies of the bridge. A one-quarter vehicle model is adopted in this study. The mass, stiffness, and damping of the vehicle are m_v_ = 7000 kg, k_v_ = 2.82 × 10^6^ N/m, and c_v_ = 390 N/m/s, respectively. The natural frequency of the vehicle is 3.20 Hz. From the above, the vehicle/bridge mass ratio is 0.042. The road surface roughness of the bridge deck is not considered in this section. The bridge and vehicle responses are obtained by solving Equation (3) using the Newmark-β method. A 500 Hz sampling rate is chosen based on the Nyquist sampling theorem to capture the dynamic response of the VBI system. The computed acceleration is infused with the white noise to simulate measurements as

(27) $${acc}_{m}={acc}_{cal}+E_{p}\times N_{\mathrm{noise}\;}\times \sigma \left({acc}_{cal}\right)$$

where ${acc}_{cal}$ represents the computed acceleration response. $E_{p}$ indicates the proportion of noise being added, and $N_{\mathrm{noise}\;}$ is a vector that follows a standard normal distribution, which means it has a mean of zero and a standard deviation of one. $\sigma \left({acc}_{cal}\right)$ is the standard deviation of the computed acceleration, determining the scale of the noise added.

**Figure 3:**
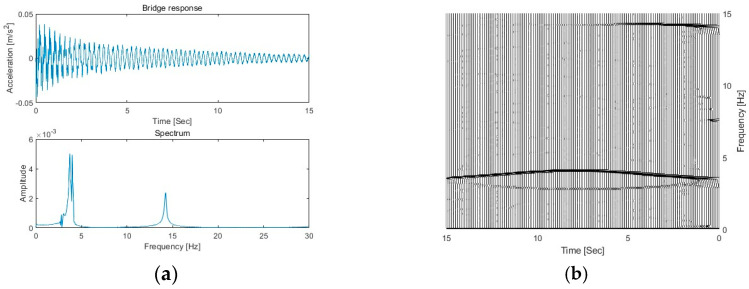
The bridge response and its TFR. (**a**) The bridge response and its Fourier spectrum; (**b**) TFR of bridge responses.

As a general case, the acceleration response at a 3/10 span of the bridge is measured, and 5% noise is added to simulate the measurement. The bridge is subject to a vehicle at a speed of 2 m/s. Figure 3a shows the acceleration response and its Fourier spectrum. From Figure 3a, there are two dominant peaks in the spectrum, which are located at 3.55 Hz and 14.10 Hz, which correspond to the first and second natural frequencies of the beam bridge.

The response data are further processed using the proposed MSST method with a window length of 2048. Figure 3b shows the TFR of the bridge response using MSST. From the figure, there are two trajectories around 3.5 Hz, with one above 3.5 Hz and another one below it. The third trajectory is around 14.1 Hz. These trajectories correspond to the IFs of the bridge response around 3.55 Hz and 14.10 Hz, respectively. In this study, the vehicle frequency is 3.2 Hz, which is smaller than the first bridge frequency of 3.55 Hz. According to Equation (6), the trajectory positioned above 3.5 Hz is the IF of the bridge, and the trajectory positioned below 3.5 Hz is the IF of the vehicle. These trajectories represent how the frequencies of the bridge and vehicle change over time, with the parabolic and concave shapes detailing their specific temporal evolution. The vehicle frequency trajectory at a lower frequency around 3.1 Hz exhibits a concave shape with the frequency decreasing before reaching a nadir and then ascending, reflecting changes in the dynamic interaction between the vehicle and the bridge. The highlight also shows that the bridge frequency component is dominant when the bridge surface is smooth.

**Figure 4:**
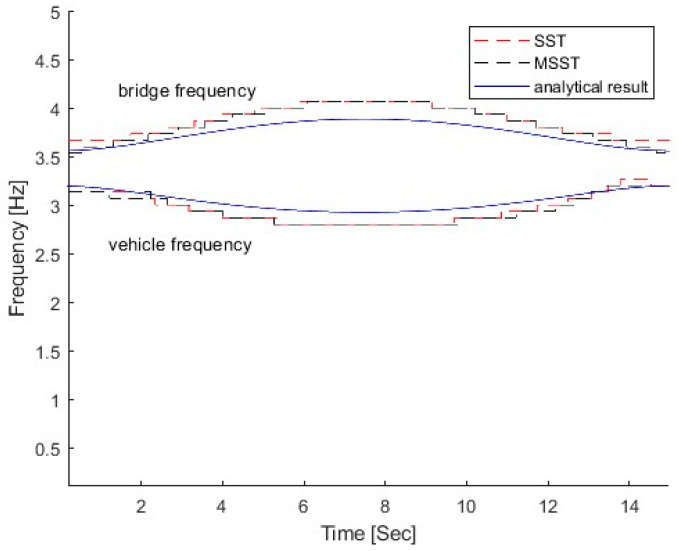
IFs of the bridge response using SST and MSST.

Figure 4 shows the IFs of the bridge response using SST and MSST, and the results are compared with the analytical solution by Equation (6). In the figure, the IF by SST, e.g., the red dashed line, displays slight fluctuations at the beginning and the end of the bridge frequency trajectory. In contrast, the result by MSST, depicted by the blue dash–dot line, shows a smoother transition throughout the bridge frequency trajectory, and it is much closer to the analytical solution compared with that by SST. The results show that MSST provides a smoother representation of time-varying characteristics, suggesting its superior ability to filter noise and capture subtle changes in the signal.

### 3.2. Parametric Analysis

In this section, the proposed MSST is used to extract the time-varying characteristics of VBI systems. The vehicle–bridge mass and frequency ratios have a significant effect on the time-varying characteristics of the vehicle–bridge interaction systems due to their coupling effect [9]. To demonstrate the performance of the proposed method, the proposed method is used to extract time-varying characteristics due to the vehicle–bridge coupling effect in this section. Except for what is specified in the sections, other parameters of the vehicle–bridge interaction system are the same as that in Section 3.1.

#### 3.2.1. Effect of the Vehicle–Bridge Mass and Frequency Ratios

The VBI system includes the vehicle and bridge subsystems, as listed in Equations (1) and (2). The interaction between the vehicle and bridge is significantly affected by the vehicle–bridge mass ratio. In this section, four vehicles with different masses are considered and their corresponding vehicle–bridge mass ratios are 0.0048, 0.00095, 0.0195, and 0.0390, e.g., Cases 1, 2, 3, and 4 in Table 1, respectively. The frequency of the vehicles is the same as 3.2 Hz, and the vehicle–bridge frequency ratio for these four cases is 0.9.

**Figure 5:**
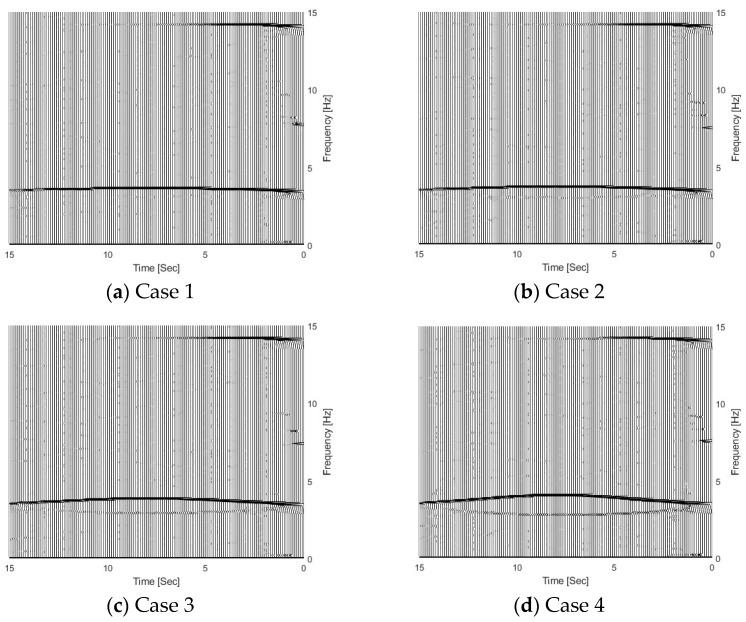
TFRs of bridge responses with different weight vehicles.

Figure 5 shows the TFRs of the bridge responses with different vehicle–bridge mass ratios, and 5% noise has been added in the calculated response. According to Equation (5), the trajectories above and below 3.55 Hz in the figure correspond to the bridge and vehicle dynamic components, respectively. From Figure 5, the bridge frequency components above 3.55 Hz are highlighted, which means that the bridge dynamic component is dominant in the dynamic responses of the bridge subjected to a moving vehicle. Comparing four cases in Figure 5, the vehicle frequency component becomes dark when the vehicle–bridge mass ratio increases. The results show that the energy of the vehicle dynamic component in the bridge response increases with the vehicle–bridge mass ratio.

**Figure 6:**
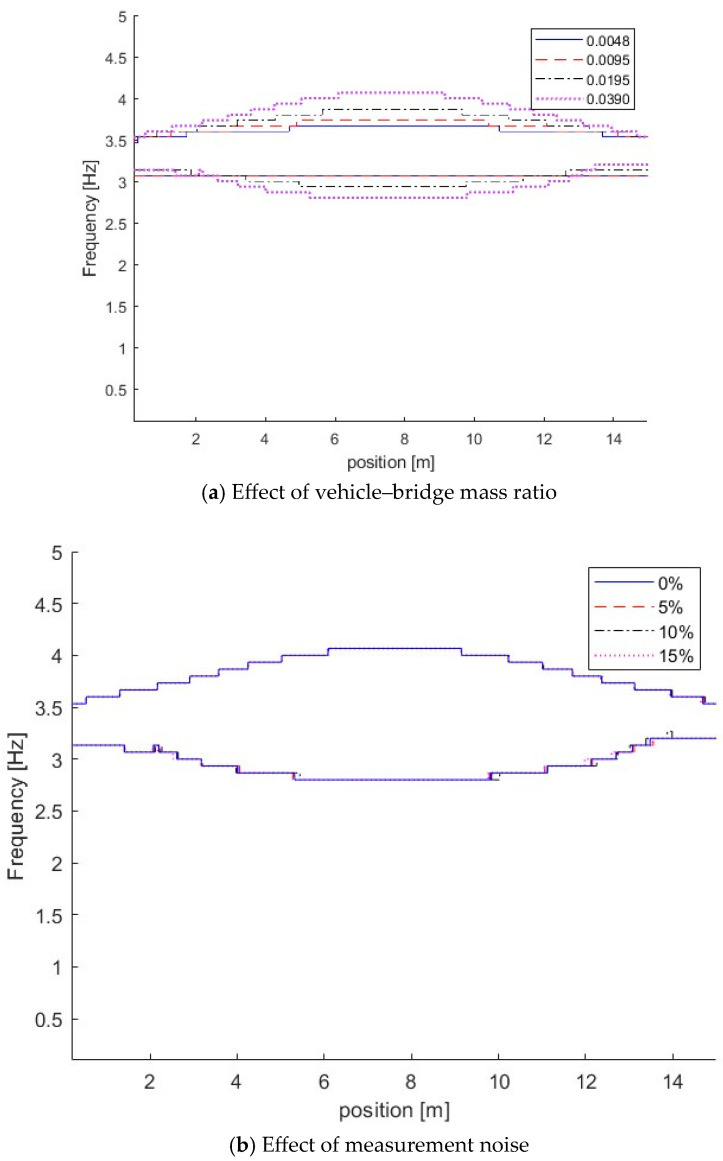
IFs of bridge responses with different vehicle–bridge mass ratios and measurement noise.

It is confirmed by their corresponding IFs of dynamic responses for the bridge with different vehicle–bridge mass ratios as shown in Figure 6a. From Figure 6a, the coupling effect between the vehicle and bridge is increased with the vehicle mass. The heavy vehicle induces the large coupling effect when the vehicle–bridge frequency ratio is the same. When the vehicle–bridge mass ratio is 0.0048, the vehicle frequency component is approximately constant. As shown above, light vehicles have a minimal impact on the dynamic characteristics of the bridge, and the bridge frequency variation is relatively small when the vehicle is passing it. Heavy vehicles significantly impact the dynamic characteristics of the bridge, and the bridge frequency variation is large.

To study the effect of measurement noise, 5%, 10%, and 15% noise were added to simulate measurements with different noise levels. Case 4 in Table 1 is studied. Figure 6b shows the IFs of Case 4 with different measurements. From Figure 6b, the IFs are close to those without measurement noise, e.g., 0% noise, and the results show that the proposed method is robust to the measurement noise. In this section, 5% noise is considered in the measurements if it is not specified.

**Figure 7:**
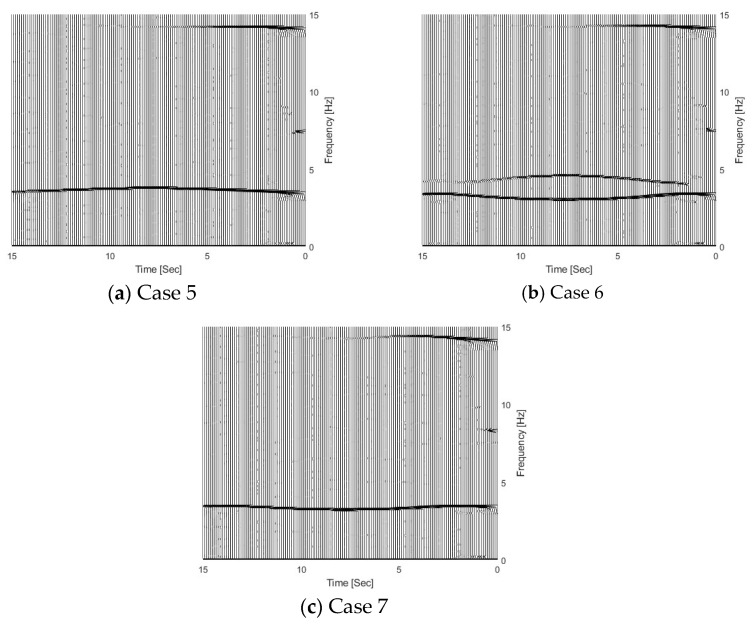
TFRs of bridge responses with different vehicle–bridge frequency ratios.

**Figure 8:**
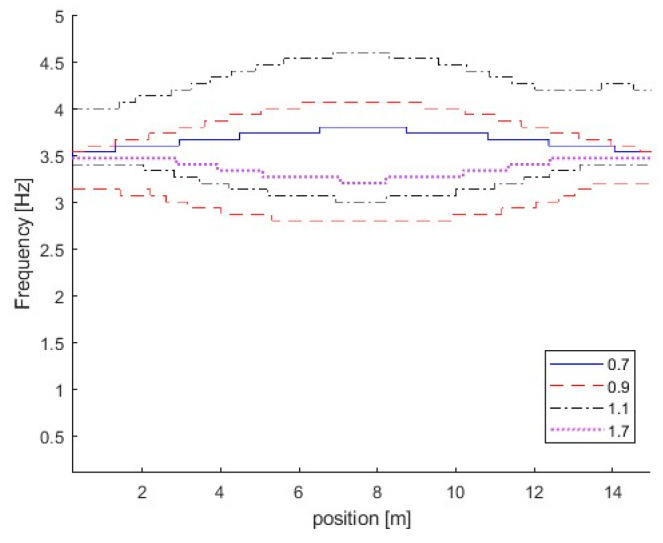
IFs of bridge responses with different vehicle–bridge frequency ratios.

**Figure 9:**
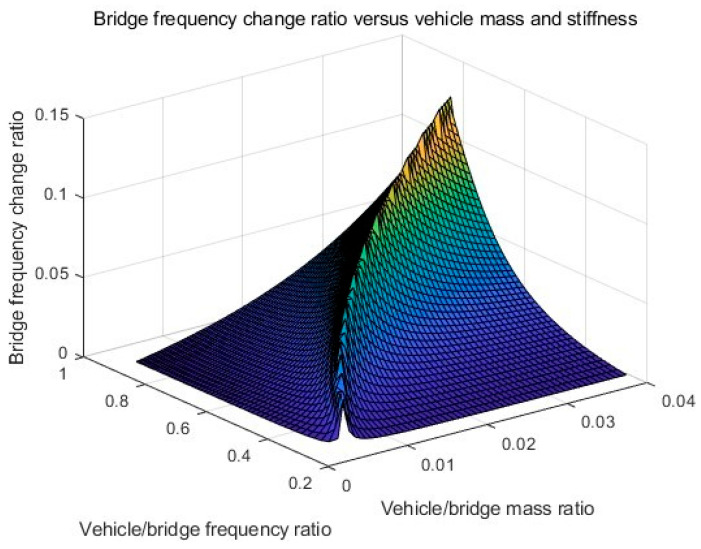
Bridge frequency change ratio versus vehicle/bridge mass and frequency ratios.

The effect of the vehicle–bridge frequency ratio is also studied in this section. The detailed parameters are listed in Table 1 as Cases 4, 5, 6, and 7. The vehicle–bridge mass ratio is the same as 0.0390 for these four cases. Figure 7 shows the TRFs of bridge responses with different vehicle–bridge frequency ratios. Figure 8 shows their corresponding IFs from the bridge responses. Figure 9 shows the relationship between the bridge frequency change ratio versus the vehicle–bridge mass and frequency ratios. For Cases 4 and 5, the vehicle frequency is smaller than the first natural frequency of the bridge at 3.55 Hz. According to Equation (6), the trajectory above 3.55 Hz is the bridge dynamic component, and the one below 3.55 Hz is the vehicle dynamic component. For Cases 6 and 7, the vehicle frequency is larger than the first natural frequency of the bridge. According to Equation (5), the trajectory above 3.55 Hz is the vehicle dynamic component and the one below 3.55 Hz is the bridge component. From Figure 7a, there are two trajectories around 3.55 Hz and 14.10 Hz for Case 5, and only the bridge frequency component around 3.55 Hz is observed. The similar result observed in Figure 7c for Case 7 and the bottom trajectory is below 3.55 Hz, corresponding to the bridge dynamic component. There are two trajectories around 3.55 Hz for Cases 4 and 6 as shown in Figure 5d and Figure 7b. The vehicle dynamic components are observed in these two cases. Comparing Cases 4 and 6, their vehicle–bridge frequency ratios are 0.9 and 1.1. The vehicle dynamic component for Case 4 in Figure 5d is below 3.55 Hz and the one for Case 6 in Figure 7b is above 3.55 Hz. The result is confirmed from their corresponding IFs in Figure 8 and the typical coupling effect between the vehicle and bridge occurs due to their vehicle–bridge frequency ratios are close to 1.0. Figure 9 provides a summary for the effect of the vehicle–bridge mass and frequency ratios on the change in the bridge frequency component. The change for the bridge frequency component increases with both the vehicle–bridge mass and frequency ratios.

#### 3.2.2. Effect of Road Surface Roughness

The dynamic response of the bridge under moving vehicles is affected by the bridge road surface roughness in Equation (3). In this section, three different road surface roughness levels are studied, e.g., Classes A, B, and C [29].

**Figure 10:**
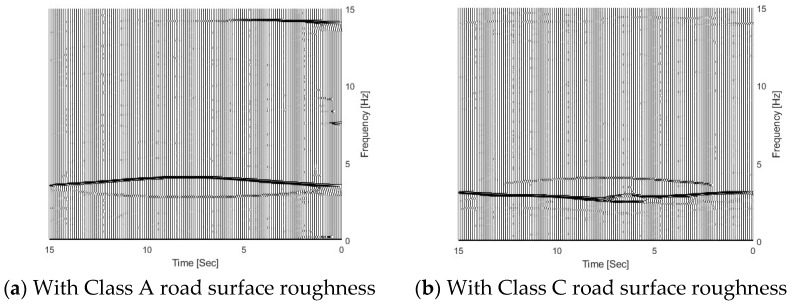
TFRs of bridge responses with different road surface roughness levels.

**Figure 11:**
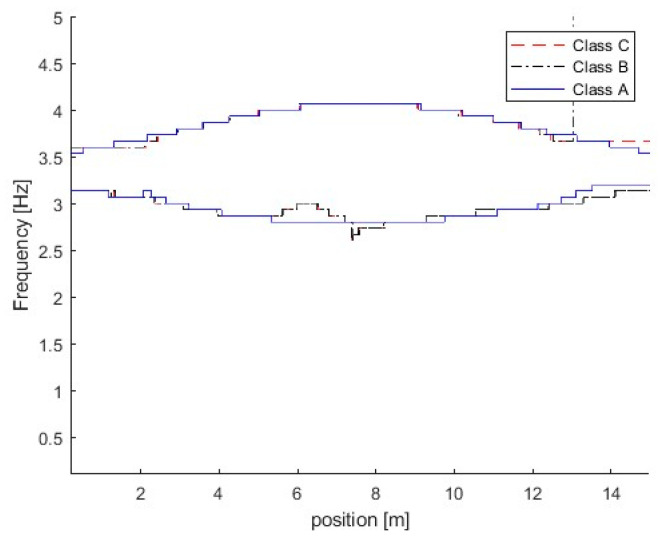
IFs of bridge responses with different road surface roughness levels.

Figure 11 shows the TFRs of the bridge responses for the bridge with different road surface roughness levels. From the figure, three trajectories are extracted successfully from bridge responses using the proposed MSST method. There are two trajectories around 3.5 Hz and the other one is around 14.1 Hz. As the vehicle frequency is 3.2 Hz and it is smaller than the bridge frequency 3.55 Hz, according to Equation (6), the trajectory below 3.5 Hz is the vehicle frequency component, and the trajectory above 3.5 Hz is the bridge frequency component. The TFR with Class A road surface roughness is shown in Figure 10a. In Figure 10a, the bridge frequency trajectory above 3.5 Hz is highlighted with a high energy, and it shows that the bridge dynamic component is dominant in the response. The TFR with Class C is shown in Figure 10b. From the figure, the vehicle frequency trajectory below 3.5 Hz is highlighted with high energy, and it shows that the vehicle dynamic component is dominant in the response. There are some oscillations in the TFRs from the bridge response with Class C road surface roughness. Figure 11 shows the IFs of the bridge responses with different road surface roughness levels. From the figure, the bridge and vehicle IFs are extracted successfully from the bridge responses with different road surface roughness levels.

#### 3.2.3. Effect of Vehicle Speed

**Figure 12:**
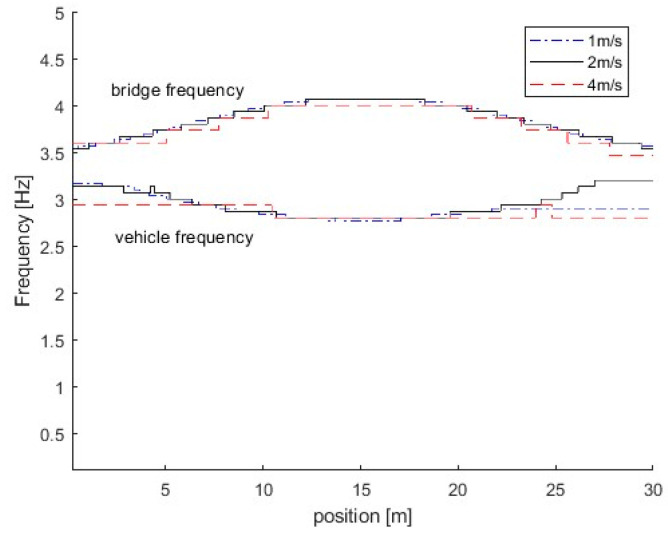
IFs of bridge responses under different vehicle speeds.

In this section, three different vehicle speeds are studied, e.g., 1 m/s, 2 m/s, and 4 m/s. Figure 12 shows the IFs from the dynamic responses of the bridge subjected to a vehicle with different speeds. From the figure, there are two trajectories around 3.5 Hz. Here, the natural frequencies of the vehicle and bridge are 3.2 Hz and 3.5 Hz, respectively. The frequency of the vehicle is smaller than that of the bridge. According to Equation (6), the trajectory above 3.5 Hz is corresponds to the IF of the bridge, and the one below 3.5 Hz is related to the vehicle. The results show that the proposed method is successful in extracting IFs from the bridge responses. In Figure 12, IFs for the case with the vehicle speed of 1 m/s are much smoother than those at a velocity of 4 m/s. This is due to the time duration for the vehicle passing over the bridge being reduced when the vehicle speed increases, and the frequency resolution being reduced. The error will be introduced on time-varying characteristics using the high vehicle speed and the low vehicle speed is recommended for bridge damage detection using the proposed method.

### 3.3. Time-Varying Characteristics of VBI Systems with Bridge Damage

The proposed method is to extract the time-varying characteristics from the dynamic responses of the bridge subjected to a moving vehicle. The bridge damage is a local phenomenon, and the local response is excited when the vehicle passes over the damage location. The local response is reflected by the time-varying characteristics, and it is very sensitive to the damage. The information of the vehicle–bridge frequency ratio is not required for structural damage detection. The change in the time-varying characteristics due to the damage is studied in this section.

In this study, the bridge damage is simulated by Equation (7). The damage extent is described by the severity parameter α and the damage range parameter β. The damage location is expressed by the parameter *l_c_*, which indicates the central point of the damage zone. Different damage scenarios are simulated using these three parameters. The time-varying characteristics of the VBI system with different damage scenarios are extracted in this section. The parameters of the VBI system are the same as those in Section 3.1.

#### 3.3.1. Effect of the Damage Severity Parameter α

**Figure 13:**
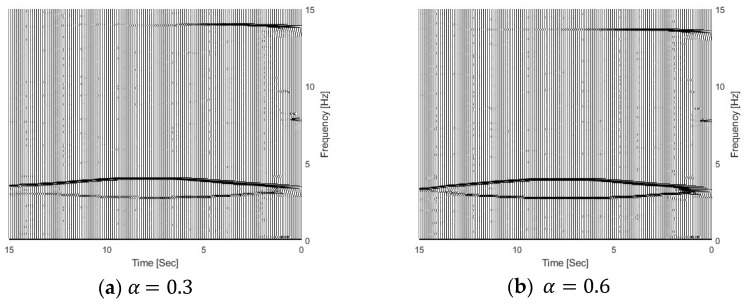
TFRs of bridge responses with different damage severities.

**Figure 14:**
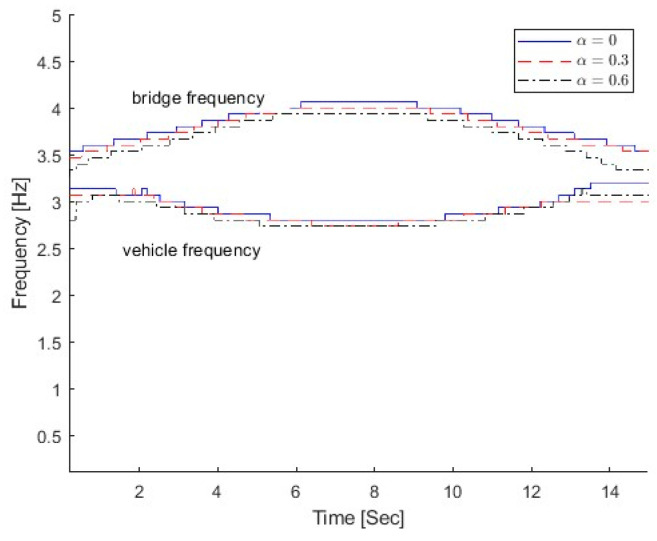
IFs of bridge responses with different α.

In this section, three different damage severities are simulated, e.g., α=0.0, 0.3, and 0.6, respectively. The damage region parameter β is 0.1, the center location of the damage zone *l_c_* is at 1/3 L, and m is 2.0. The natural frequencies of the bridge with different damage scenarios are 3.55 Hz, 3.48 Hz, and 3.38 Hz, respectively, and their corresponding vehicle–bridge frequency ratios are 0.90, 0.92, and 0.95. Figure 13 shows the TFRs of the bridge responses with different bridge damage α=0.3 and 0.6, and Figure 3b shows the TFR without the damage, e.g., α=0.0. Figure 10 shows their corresponding IFs. In Figure 3b and Figure 13, three time-varying frequency trajectories are clearly observed. There are two trajectories around 3.0~3.5 Hz and another trajectory around 14.0 Hz. As shown in Figure 4b, there are two trajectories around 3.5 Hz and another trajectory around 14.1 Hz. The trajectory above 3.5 Hz corresponds to the bridge frequency component as listed in Equation (6), and the highlight shows that the bridge frequency component is dominated in the response. The trajectory below 3.5 Hz is related to the vehicle frequency component. The trajectory around 14.1 Hz is related to the second bridge frequency component. In Figure 13, the trajectories are moving downward when the damage severity parameter α increases and the bottom trajectory also becomes dark. The results show that the energy of the vehicle frequency component increases when the bridge frequency approaches the vehicle frequency. When α=0.6, the first natural frequency of the bridge is 3.38 Hz and that is close to the vehicle frequency of 3.2 Hz. Both trajectories around 3.0 Hz are highlighted and the signal energy is distributed to both the vehicle and bridge dynamic components. The results show that the pattern change in TFRs could be used to indicate structural damage. The results are also confirmed by the IFs in Figure 14. The IFs move downward when the damage severity increases.

As shown above, the proposed method is effective and accurate to extract the time-varying characteristics from the dynamic responses of the bridge subjected to a moving vehicle. The change pattern of the time-varying characteristics could be a potential indicator for structural damage. However, there is still a big challenge to use the time-varying characteristics for bridge structural damage detection due to the uncertainty, such as road surface roughness and operational varieties. A previous study successfully utilized the time–frequency domain features of the vehicle–bridge interaction systems for structural damage detection using transfer learning and continuous wavelet transform [30]. Further study needs to be conducted for practical applications considering complex vehicle–bridge interaction systems using machine learning models.

#### 3.3.2. Effect of Damage Region Parameter β

The parameter β represents the range of the damaged region, which is another parameter that affects the damage extent. β is a dimensionless parameter, which is defined as the ratio between the length of the damage region and the total length of the bridge. In this section, three different damage regions representing no damage, small damage, and large damage are studied, e.g., β=0.0, 0.1, 0.2. Other parameters are α=0.3, *l_c_* = 1/3 L, and m = 2.0. The first bridge’s natural frequencies for three damage scenarios are 3.55 Hz, 3.44 Hz, and 3.25 Hz, respectively.

**Figure 15:**
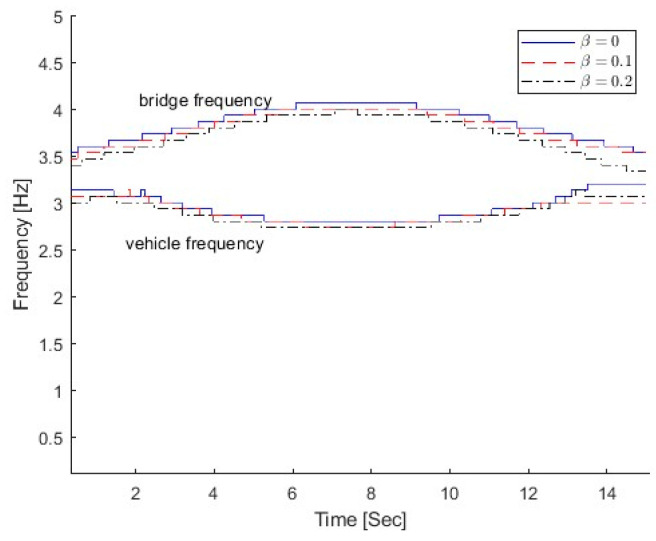
IFs of bridge response with different β.

Figure 15 shows the IFs from the dynamic responses of the bridge with different damage parameters β. For all damage scenarios, the first bridge frequency is larger than the vehicle frequency. So the top IFs correspond to the bridge frequency component and the bottom ones relate to the vehicle frequency component. As seen in the figure, the IFs move downward when the damage parameter β increases.

#### 3.3.3. Effect of the Damage Location

**Figure 16:**
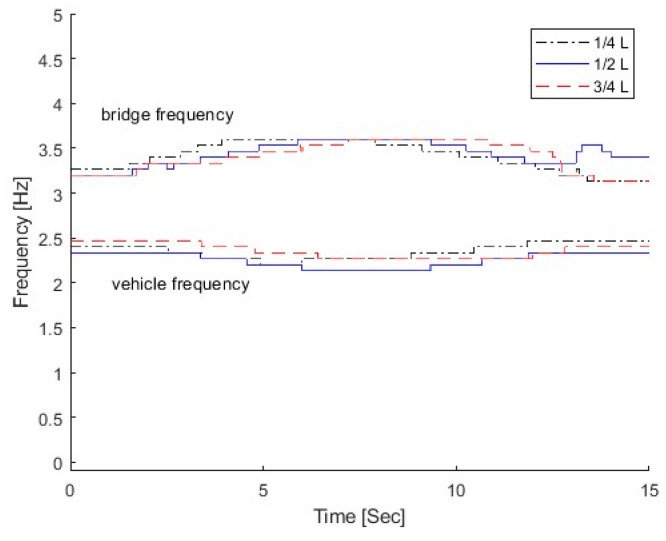
IFs of bridge responses with different damage locations.

In this section, three damage locations are studied, e.g., $l_{c}=\frac{1}{4}L,\frac{1}{2}L,\frac{3}{4}L.$ Other damage parameters are α=0.3, β=0.1, and m = 2.0. The parameters of the VBI system are the same as those in Section 3.1. In total, 5% is added to simulate measurements. Figure 16 shows the IFs from the dynamic responses of the bridge with different damage locations. The same as in Section 3.3.2, the top IFs correspond to the bridge frequency component and the bottom ones relate to the vehicle frequency component. From the figure, there is a large variation in the bridge frequency component at the damage location. For the case with the damage at 1/4 L, the bridge IF exhibits a frequency variation at 1/4 L. The frequency variation is observed around 3/4 L for the case with the damage at 3/4 L. This is due to the local response being excited when the vehicle is passing over the damage location. The local response is very sensitive to the damage. The local frequency variation could be used to localize the damage.

## 4. Experimental Study

### 4.1. Experimental Setup

**Figure 17:**
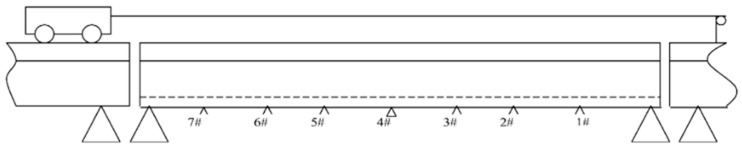
Experimental setup.

A vehicle–bridge interaction experimental system was built in the laboratory. Figure 17 shows the experimental setup [9]. The bridge model includes three Tee-section reinforced concrete beams, e.g., the 4.5 m long leading beam, the 5 m long main beam and the 4.5 m long tailing beam. The leading beam is used for the vehicle to accelerate, and the tailing beam is used for the vehicle to decelerate. The gaps between the beams are 10 mm. The vehicle is pulled along the beam by an electrical motor at an approximate speed of 0.5 m/s. Seven accelerometers are installed evenly at the bottom along the beam to monitor the acceleration responses. These seven evenly distributed accelerometers along the length of the bridge are mainly for modal testing. The proposed method is to extract time-varying characteristics from the dynamic responses of the bridge subjected to a moving vehicle. The vehicle is a moving excitor and only one single sensor is needed. In this study, the accelerometer at a 3/8 span of the bridge is used. The single sensor is arbitrarily chosen and the results from the other accelerometers will be similar. The sampling frequency is 2024.292 Hz and the time duration for each test is 30 s.

**Figure 18:**
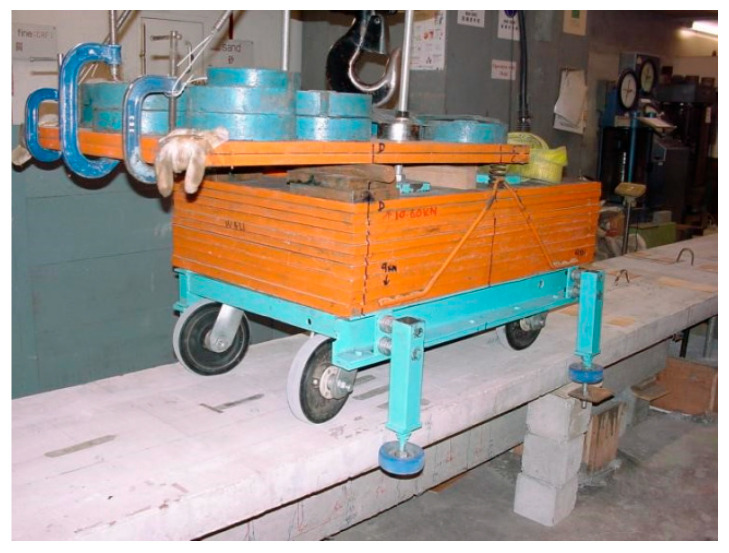
The vehicle system on the bridge.

Figure 18 shows the vehicle model on the bridge. The axle spacing of the vehicle is 0.8 m. Two vehicles with different weights are simulated in this study. For the light vehicle, the total weight is 10.60 kN, and the front and rear axle loads are 5.58 kN and 5.02 kN, respectively. For the heavy vehicle, the total weight is 15.06 kN, and the front and rear axle loads are 7.92 kN and 7.14 kN, respectively.

Two damage scenarios in the main beam were created for this study. The three-point load system is used to create damage. The small damage was created by adding the static load at 1/3 L from the left support. The load gradually increased up to 50 kN at 2 kN increments, and a damage zone was created with a visual crack depth of 213 mm at the load position and a crack zone 760 mm wide. The load was kept for 30 mins, and then the static load was removed. For the damage scenario, the same beam was loaded at 2/3 L of the beam up to 50 kN using the three-point load system. A further static test was conducted using the four-point load system with loading at 1/3 L and 2/3 L. The large damage scenario was created with the final load 105 kN without yielding the main reinforcement [9]. The largest crack depth was 281 mm around the middle of the beam and the crack zone was 2371 mm wide. The bridge with these three cases, e.g., undamaged, small, and large damage, is studied with the vehicle passing over the bridge. The dynamic responses of the bridge subjected to the vehicle with different weights are analyzed using the proposed MSST.

### 4.2. Effect of the Vehicle Weight

**Figure 19:**
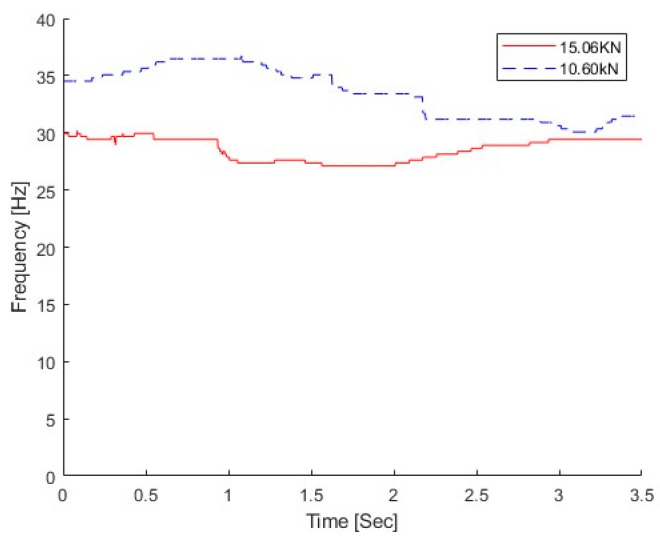
IFs of bridge responses without damage.

The vehicle with two different weights was studied in this section, e.g., 10. 60 kN and 15.06 kN. Compared with the total mass of the concrete beam of 1050 kg, the mass ratios between the vehicle and bridge are 1.01 and 1.43 for those two vehicles, respectively. The dynamic responses at the middle of the bridge subjected to the moving vehicle are used. The vehicle speed is 0.5 m/s. There is no damage to the bridge, and the natural frequency of the bridge without damage is 30.69 Hz from the modal testing [9]. Figure 19 shows the IFs extracted from the dynamic responses of the bridge under different weight vehicles using the proposed MSST. From the figure, the IF under the light vehicle is much larger than that under the heavy vehicle. This result indicates that the proposed method could successfully extract the time-varying characteristics of the bridge under a moving vehicle.

### 4.3. IFs of the Bridge with Different Damage Scenarios

**Figure 20:**
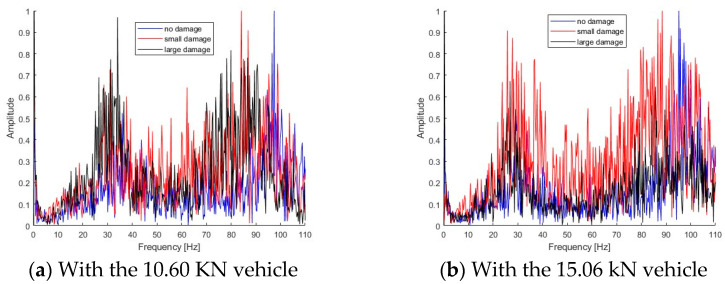
Fourier spectra of bridge responses subjected to different vehicles.

Small and large damage scenarios have been created using the static loading as shown in Section 4.1. Figure 20 shows the Fourier spectra of dynamic responses for the bridge under a moving vehicle. Figure 20a shows the Fourier spectra of bridge responses with different scenarios subjected to a light vehicle, and Figure 20b shows the spectra with a heavy vehicle. As seen in Figure 20, there are two peaks in the spectra and they are around 30 Hz and 95 Hz.

**Figure 21:**
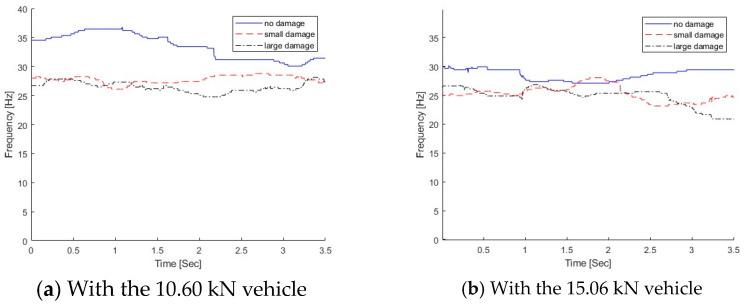
IFs of dynamic responses from the bridge under different vehicles.

The proposed MSST method is used to extract the time-varying characteristics from the dynamic responses of the bridge subjected to a moving vehicle, and the results are shown in Figure 21. Figure 21a shows the IFs of the bridge with different damage levels when a light vehicle is passing over it, and the IFs with the passage of the heavy vehicle are shown in Figure 21b. From Figure 21a, the overall IF reduces as the damage increases. For the small damage scenario, compared with the right region without damage, the bridge IF is much lower when the vehicle is passing the crack damage zone around 1/3 L of the beam from the left support. For the large damage scenario, the bridge IF is lower over the wide crack damage zone. The same observation could be observed in Figure 21b with a heavy vehicle. For the small damage scenario, the frequency ranges between 28 and 30 Hz under the light vehicle, and it is between 24 and 26 Hz under the heavy vehicle. For the large damage scenario, the frequency range reduces to 24–26 Hz under the light vehicle, and it is 22–24 Hz under the heavy vehicle.

In practice, the modal testing is normally conducted under ambient conditions, and the bridge needs to be closed. In operational environments, the frequency change due to damage may be much smaller than that due to operational and environmental variations. From the modal testing, the first natural frequencies of the bridge with no damage, small damage, and large damage are 30.69 Hz, 27.02 Hz, and 25.7 Hz, respectively [9]. In this study, the proposed method is used to extract the time-varying characteristics from the dynamic responses of the bridge subjected to a moving vehicle. As shown in Figure 21, the frequency variation with the passage of the vehicle for the light vehicle is between 28 and 30 Hz for small damage and that for the heavy vehicle is between 24 and 26 Hz. The frequency variation due to damage is at the same level as that due to the passage of the vehicle. The damage cannot be detected by the frequency change only. A previous study has also shown that the vehicle passing over the damage zone would induce the local nonlinear response due to the crack being open or closed [9]. The change in the dynamic responses of the bridge due to damage could be amplified by the presence of the vehicle on top. The frequency change pattern is a potential indicator for structural damage. Leveraging the time-varying features, further study will be conducted for structural damage detection using machine learning models.

## 5. Conclusions

A new MSST-based method was developed to extract the time-varying characteristics for the structural damage detection of the bridge subjected to moving vehicles. The numerical and experimental studies were conducted to show the performance of the proposed method to extract time-varying characteristics for bridge damage detection. Some conclusions were obtained, as shown below:

(1) Numerical results of the time-varying signal analysis show that the proposed MSST method can obtain a higher energy concentration and clearer time–frequency representation than that by SST. It is effective and accurate to extract the time-varying features of non-stationary signals.
(2) Numerical and experimental results show that the proposed MSST method is effective and accurate in extracting the time-varying features of the vehicle–bridge interaction system. When the vehicle–bridge frequency ratio is smaller than 1, the bridge frequency component will be dominated in the time–frequency representation of the bridge responses.
(3) From numerical and experimental results, the IF is reduced as the bridge damage increases. The local response is excited when the vehicle is passing over the damage location. The local variation in the IF at the damage location could be used to indicate the damage location. The change in the IF pattern is a good indicator of the bridge damage.
(4) The vehicle speed affects the performance of the proposed MSST method to extract the time-varying characteristics and the low vehicle speed is recommended for bridge damage detection.
(5) The performance of the proposed method to detect the damage zone of reinforced concrete bridges is validated numerically and experimentally. Further study needs to be conducted for practical applications, considering complex vehicle–bridge interaction systems using machine learning models.

## Figures and Tables

**Table 1:** Parameters of different vehicles.

Case	Stiffness (N/m)	Mass of Vehicle (kg)	Frequency of Vehicle (Hz)	Vehicle/Bridge Mass Ratio	Vehicle/Bridge Frequency Ratio
1	3.53 × 10^5^	875	3.20	0.0048	0.90
2	7.05 × 10^5^	1750	3.20	0.0095	0.90
3	1.41 × 10^6^	3500	3.20	0.0190	0.90
4	2.82 × 10^6^	7000	3.20	0.0390	0.90
5	1.71 × 10^6^	7000	2.49	0.0390	0.70
6	4.23 × 10^6^	7000	3.91	0.0390	1.10
7	1.01 × 10^7^	7000	6.04	0.0390	1.70

## Data Availability

The data that support the findings of this study are available on request from the corresponding author.
